# Reply to Das and Berkhout, “How Polypurine Tract Changes in the HIV-1 RNA Genome Can Cause Resistance against the Integrase Inhibitor Dolutegravir”

**DOI:** 10.1128/mBio.00623-18

**Published:** 2018-05-29

**Authors:** Isabelle Malet, Frédéric Subra, Clémence Richetta, Charlotte Charpentier, Gilles Collin, Diane Descamps, Vincent Calvez, Anne-Geneviève Marcelin, Olivier Delelis

**Affiliations:** a Sorbonne Université, INSERM, Institut Pierre Louis d’Epidémiologie et de Santé Publique (iPLESP), AP-HP, Hôpital Pitié Salpêtrière, Laboratoire de Virologie, Paris, France; b LBPA, ENS Cachan, CNRS UMR 8113, IDA FR3242, Université Paris-Saclay, Cachan, France; c IAME, UMR1137, INSERM, Université Paris Diderot, Sorbonne Paris Cité, AP-HP, Hôpital Bichat, Laboratoire de Virologie, Paris, France; Medical School, University of Athens

**Keywords:** 1-LTR, 3' PPT, HIV-1, dolutegravir, integration

## REPLY

Strand transfer inhibitors are potent molecules targeting HIV-1 integration, a critical step involved in retroviral replication. To date, three inhibitors, raltegravir, elvitegravir, and dolutegravir (DTG), are available to treat infected patients. Unfortunately, many resistance pathways have been described for raltegravir and elvitegravir, and all mutations leading to strand transfer resistance have been located in the integrase gene. In the case of dolutegravir, the last integrase inhibitor, only a few mutations described from patients or from *in vitro* selection are reported to confer resistance (1–3).

Following the publication of the article describing a virus having selected mutations outside the integrase gene and conferring resistance to HIV-1 integrase inhibitors (4), Das and Berkhout propose a model for the replication of this 3′-polypurine-tract (3′-PPT)-mutated virus (18). Integration of the viral DNA would be possible due to the modification of the 5′ long terminal repeat (LTR) end which makes the virus insensitive to dolutegravir action, allowing it to integrate into the host genome. This hypothesis is plausible since an optimal binding of dolutegravir on the integrase/DNA complex is required for inhibition of the compound and the binding requires the canonical LTR end (5). Since integration is a concerted mechanism occurring in the intasome composed of integrase and both LTR ends, the deficient binding of dolutegravir on the 5′ LTR could allow the unmodified 3′ LTR end to become insensitive to dolutegravir. Indeed, as reported in the literature, mutations at one end could have consequences for the other end (6). Consequently, integration of both 5′ LTR and 3′ LTR could occur despite lower efficiency, explaining the resistance of the 3′-PPT mutant to dolutegravir.

**FIG 1:**
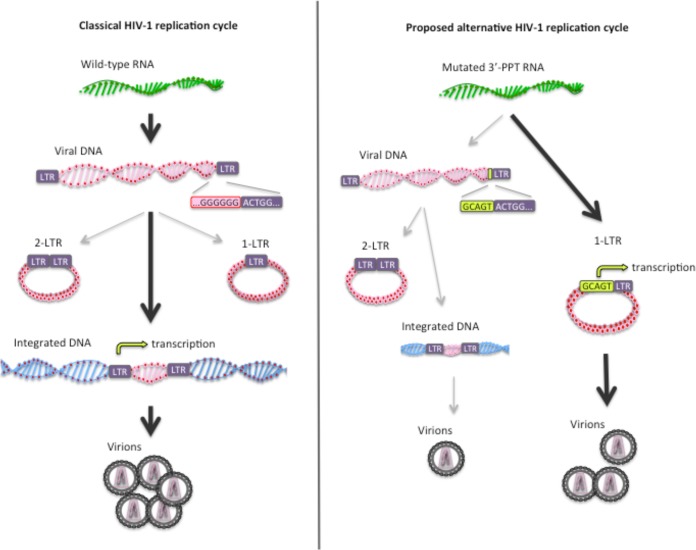
Comparison between the classical HIV-1 replication cycle and the proposed alternative replication cycle of the 3′-PPT mutant. (Left) The classical HIV-1 replication cycle leading to the integration of viral DNA at the level of genomic DNA. (Right) The proposed alternative replication cycle leading mainly to the formation of 1-LTR circles during the reverse transcription steps. The CAGT sequence inside the mutated 3′ PPT, upstream of U3, could be an enhancer of transcription initiation, allowing viral production from 1-LTR circles and explaining the resistance to DTG.

Since 2-LTR circles are formed by ligation of the two LTR ends by the nonhomologous end joining (NHEJ) pathway, the LTR-LTR junction should reflect the integrity of the LTR ends (7). To test this hypothesis, sequencing of the U3-U5 junction of 2-LTR circles was carried out from MT-4 cells infected with the wild-type (WT) and 3′-PPT-mutated viruses with or without DTG (without DTG at day 6 for WT and day 8 for mutated virus and with DTG at day 15 for 3′-PPT-mutated virus). Briefly, after extraction of DNA, a fragment of 307 nucleotides encompassing more than 100 nucleotides from either side of the junction was amplified using primers specific for U3 and U5 sequences, and pyrosequencing on a GS Junior sequencer (Roche 454 Life Sciences) was performed. A total of 1,675 and 1,280 reads per nucleotide position was amplified for WT and mutant viruses, respectively. Interestingly, we found that the LTR-LTR junction was identical to the palindromic sequence found by the predicted ligation of the two unprocessed DNA ends with a total similitude between the two analyzed viruses. Sequence analysis, as described in the literature (17), showed that around 50% (53.7% for WT and 50% for the mutant) of circle junctions were similar to the expected sequence and that the remaining 50% of sequences had multiple deletions ranging from 1 to several tens of nucleotides, located on either side of the junction (U5 or U3) or extending over the U5-U3 junction. These data show that no additional bases were detected in the 3′ PPT compared to the WT virus, highlighting the classical DNA ends of the 2-LTR WT virus. These data sustain the idea that the reverse transcription step does not lead to a modification of LTR ends during the replication of the 3′-PPT-mutated virus (Fig. 1).

**FIG 2:**
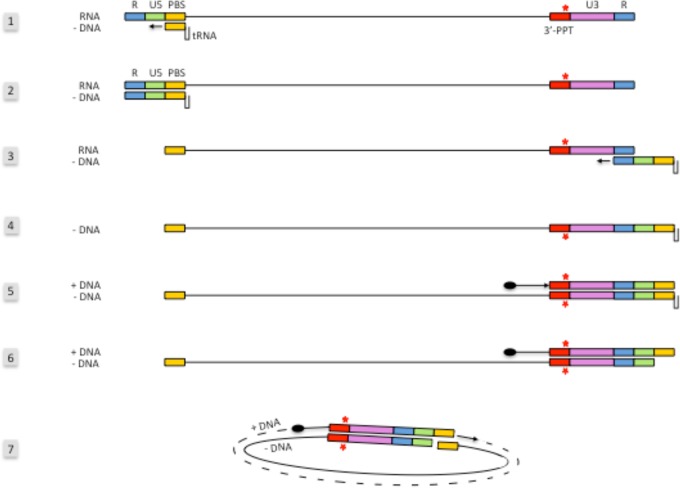
Schematic representation of the reverse transcription process of the 3′-PPT mutant leading to the 1-LTR circle formation. The first reverse transcription steps of the 3′-PPT mutant (steps 1 to 3) are similar to the WT. In step 4, we hypothesize that the entire RNA sequence, including the mutated PPT, is degraded by RNase H and that the initiation of the +DNA synthesis (step 5) occurs from sequences located upstream of the 3′ PPT. Once the partial +DNA is synthesized and the RNase H has cleaved the tRNA^Lys^ (step 6), the partially double-stranded DNA can be circularized by pairing the PBS sequences located on both sides of the DNA, which leads to the formation of 1-LTR circles.

We agree with Das and Berkhout in suggesting that the mutation in the 3′ PPT leads to a modification of the reverse transcription, but instead of modifying LTR ends, we predict for ourselves that the presence of the mutation, disrupting the 3′ PPT, would lead to a total degradation of the RNA by the RNase H activity and an impairment of the synthesis of the U3-R-U5-PBS (primer binding site) +DNA from the 3′ PPT (Fig. 2). The initiation of the +DNA could be then started from sequences located upstream of the 3′ PPT as reported in the literature (8). We can speculate, in this case, that synthesis of a longer +DNA fragment could prevent the translocation of the DNA that would lead to an intramolecular circularization of the viral genome using the complementary PBS regions. After circularization, complementation of the DNA could occur and lead to the circularized genome becoming a 1-LTR circle. It can be noted that Kantor and colleagues have previously shown that mutations in the 3′ PPT lead to an accumulation of 1-LTR circles and a significant decrease of the linear viral DNA from reverse transcription. This observation, made in the context of viral vector, reinforces our hypothesis (9), an assumption that we can test by quantification of 1-LTR circles during infection with the 3′ PPT mutant.

The role of unintegrated viral DNA in HIV-1 expression has always been a topic of debate. First, it has been clearly described that unintegrated viral DNA could be involved in expression of viral accessory proteins such as Nef (10). Second, even if unintegrated viral DNA could hardly be at the origin of the production of infectious viral particles, recent reports describe a role in HIV-1 replication under specific conditions (11).

We hypothesize that, in the case of an HIV-1 genome exclusively present in unintegrated forms, as suggested for the 3′-PPT mutant, the LTR-mediated transcription of the unintegrated viral DNA of this virus should then be stronger than in the case of the wild-type unintegrated viral DNA under DTG.

Interestingly, we can notice in the 3′-PPT mutant the presence of the CAGT pattern just upstream of the 3′ LTR which has been described to be an initiator element for transcription in some viruses, such as baculovirus (12). This sequence needs to be studied for its ability to improve the transcription of the nonintegrated viral DNA of the 3′-PPT mutant, which could then support the hypothesis that the 3′-PPT mutant virus is able to replicate without going through the integration but at a lower level than the wild-type virus.

It is well known that a significant proportion of patients failing on integrase inhibitors and even more when failing on dolutegravir do not select any mutations in the integrase gene (13–15). It is difficult to know precisely why these patients failed on treatment, but depending on the context, it could be explained by the presence of unknown integrase resistance mutations, problems of adherence, and so on. The presence of selected mutations in the 3′ PPT could be also considered, as it has not been explored yet in these patients. In addition, Wijting et al. recently reported (16) the case of a patient who failed a monotherapy treatment with dolutegravir in the DOMONO study without selecting a mutation in the integrase gene but who showed mutations in the 3′ PPT similar to those reported by Malet and colleagues (4).

Taken together, these data could suggest a role of unintegrated viral DNA that would allow a significant replication of the virus, in a way independent of integration. To study this hypothesis, quantifications of integrated and nonintegrated viral DNA will be performed from cells infected with the 3′-PPT virus, and also, the CAGT pattern will be studied for its ability to increase viral transcription of the 3′-PPT mutant.
